# Automatic text classification of actionable radiology reports of tinnitus patients using bidirectional encoder representations from transformer (BERT) and in-domain pre-training (IDPT)

**DOI:** 10.1186/s12911-022-01946-y

**Published:** 2022-07-30

**Authors:** Jia Li, Yucong Lin, Pengfei Zhao, Wenjuan Liu, Linkun Cai, Jing Sun, Lei Zhao, Zhenghan Yang, Hong Song, Han Lv, Zhenchang Wang

**Affiliations:** 1 Department of Radiology, Beijing Friendship Hospital, Capital Medical University, No. 95 YongAn Road, Beijing, 100050 People’s Republic of China; 2 School of Biological Science and Medical Engineering, Beihang University, No.37 XueYuan Road, Beijing, 100191 People’s Republic of China; 3 School of Computer Science and Technology, Beijing Institute of Technology, No. 5, South Street, Zhongguancun, Haidian District, Beijing, 100050 People’s Republic of China; 4 School of Medical Technology, Beijing Institute of Technology, No.5 Zhongguancun East Road, Beijing, 100050 People’s Republic of China

**Keywords:** Artificial intelligence, Natural language processing, Deep learning, Radiology report, Bidirectional encoding representation of transformer

## Abstract

**Background:**

Given the increasing number of people suffering from tinnitus, the accurate categorization of patients with actionable reports is attractive in assisting clinical decision making. However, this process requires experienced physicians and significant human labor. Natural language processing (NLP) has shown great potential in big data analytics of medical texts; yet, its application to domain-specific analysis of radiology reports is limited.

**Objective:**

The aim of this study is to propose a novel approach in classifying actionable radiology reports of tinnitus patients using bidirectional encoder representations from transformer BERT-based models and evaluate the benefits of in domain pre-training (IDPT) along with a sequence adaptation strategy.

**Methods:**

A total of 5864 temporal bone computed tomography(CT) reports are labeled by two experienced radiologists as follows: (1) normal findings without notable lesions; (2) notable lesions but uncorrelated to tinnitus; and (3) at least one lesion considered as potential cause of tinnitus. We then constructed a framework consisting of deep learning (DL) neural networks and self-supervised BERT models. A tinnitus domain-specific corpus is used to pre-train the BERT model to further improve its embedding weights. In addition, we conducted an experiment to evaluate multiple groups of max sequence length settings in BERT to reduce the excessive quantity of calculations. After a comprehensive comparison of all metrics, we determined the most promising approach through the performance comparison of F1-scores and AUC values.

**Results:**

In the first experiment, the BERT finetune model achieved a more promising result (AUC-0.868, F1-0.760) compared with that of the Word2Vec-based models(AUC-0.767, F1-0.733) on validation data. In the second experiment, the BERT in-domain pre-training model (AUC-0.948, F1-0.841) performed significantly better than the BERT based model(AUC-0.868, F1-0.760). Additionally, in the variants of BERT fine-tuning models, Mengzi achieved the highest AUC of 0.878 (F1-0.764). Finally, we found that the BERT max-sequence-length of 128 tokens achieved an AUC of 0.866 (F1-0.736), which is almost equal to the BERT max-sequence-length of 512 tokens (AUC-0.868,F1-0.760).

**Conclusion:**

In conclusion, we developed a reliable BERT-based framework for tinnitus diagnosis from Chinese radiology reports, along with a sequence adaptation strategy to reduce computational resources while maintaining accuracy. The findings could provide a reference for NLP development in Chinese radiology reports.

**Supplementary Information:**

The online version contains supplementary material available at 10.1186/s12911-022-01946-y.

## Introduction

The overall prevalence of tinnitus among the general population ranges from 10% to 14.5% [1, 2], and 30% of people with tinnitus report ‘moderate’ to ‘very big’ difficulties in daily life [3]. There are a variety of conditions that can cause tinnitus, such as jugular bulb diverticulum, acoustic neuroma or defect of bone plate in sigmoid sinus. Medical imaging is one of the most common tools for detecting the presence of tinnitus. However, as radiology reports offer a comprehensive description of visible lesions, lesions related to tinnitus account for only a small proportion compared with commonly reported degeneration or chronic lesions [4]. Especially in elderly patients, physicians may fail to notice such findings for many reasons, such as lack of experience in diagnosis, moreover, classifying findings correlated with tinnitus requires high expertise in ENT radiology [^5^].

Hence, an automatic identification tool for actionable reports is needed, so that physicians achieve better decision making without spending extra time on selecting appropriate patients from massive radiology reports. Thus, it is challenging as well as attractive to develop an automated approach of accurately classify the actionable reports.

Radiology reports are constructed with domain-specific terms and patterns, and most of them contain unstructured data [6]. The typical format of a free-text radiology report consists of four sections: the demographics section describes basic information such as the patient’s name, age, gender, etc., the clinical information section refers to the medical history or current syndrome. The imaging findings section is the main body of the report which uses anatomic, pathologic, and radiologic terminology to describe all the normal and abnormal findings within the scanning field. Finally, the Impression section includes specific diagnosis or differential diagnosis by the radiologist,an example of a Chinese radiology report used in this study is shown in Additional file 1: FigureS1. The written style of reports varies among radiologists, and they could contain a number of literature errors [7, 8]. Manual classification of actionable reports from a large database is time-consuming, error-prone, and requires experience to rectify possible errors [9]. Despite the use of prompting for structured reporting, free-text radiology reports are still favored for their flexibility and low cost in major hospitals [10]; this trend necessitates the application of modern informatics to improve the effectiveness of radiology reports in clinical workflow and biomedical research.

**Fig. 1:**
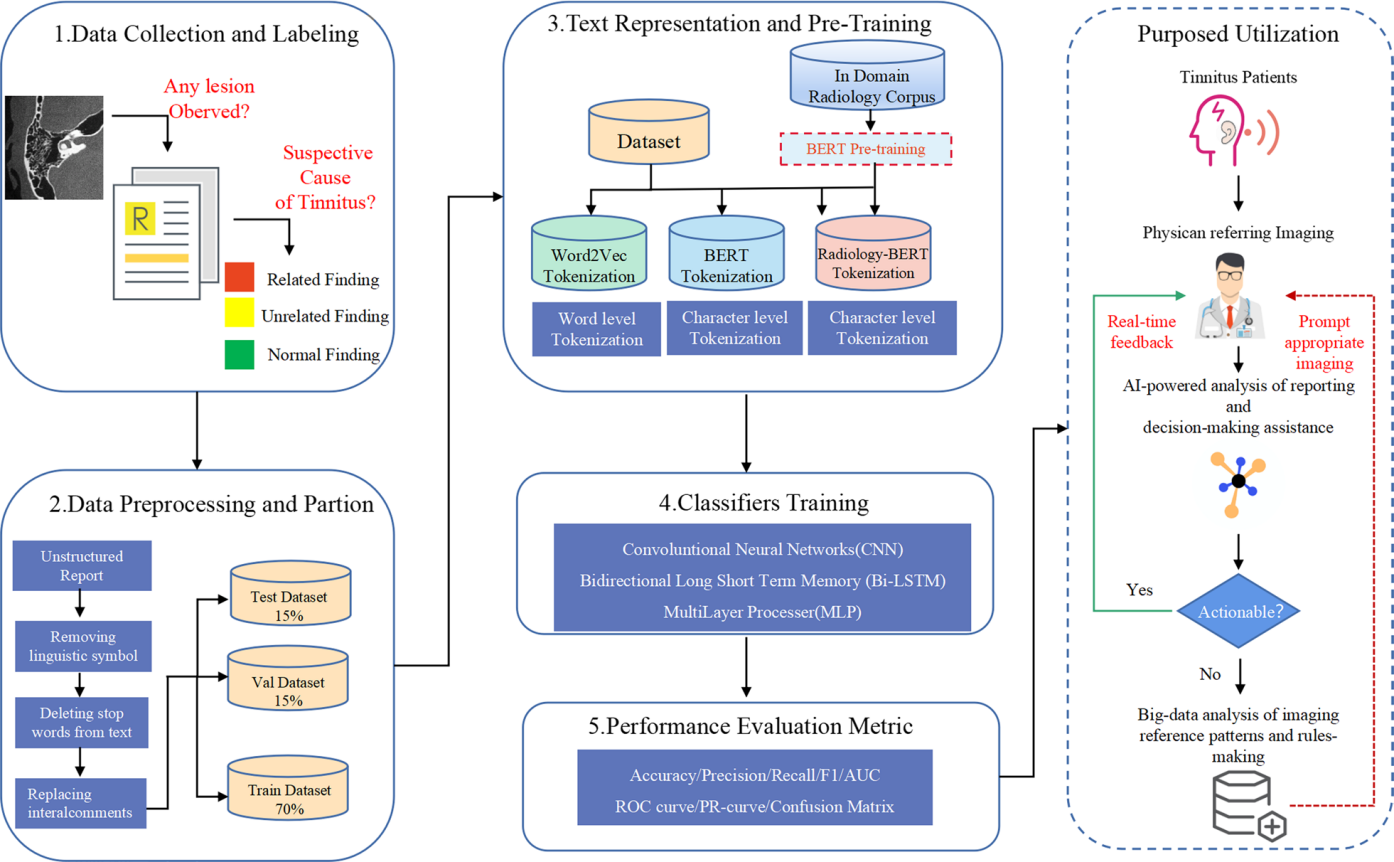
Workflow of study

Natural language processing (NLP) techniques have introduced a new era for free-text analysis and data mining [11, 12]. Traditional symbolic and statistical NLP methods may perform well on questions that can be defined exactly by a certain set of rules or decomposed simply with statistical patterns of terms in a document; both of them have good results in research cases, including data mining of radiology reports [13, 14]. Deep learning methods with modified neural networks achieved state-of-the-art results with simplicity, flexibility, and task specificity on large-scale complex tasks [15–17]. The convolutional neural network (CNN) and recurrent neural network (RNN) framework has been widely used in classification tasks due to their distinguishing performance in representation learning. CNNs can capture features between consecutive words and shift-invariant classification of input information according to its hierarchical structure. The RNN framework has gained attention for its ability to deal with variable-length input and output [18]; yet, RNNs typically show poor performance when dealing with long sequences due to the gradient vanishing and exploding problem. For this problem, a variant of RNN named long short-term memory (LSTM) network has been developed through controlling the weight of previous inputs by adding and regulating “gates”; the gates act as controllers to enable the network to retain long-range connections in training.

Apart from RNN-based models, self-attention based transformer models have gained much attention in providing more feasible representations by forming more dense correlations between words in a sequence [19]. In contrast to RNNs that rely on constructing the relationship between words in a sequential manner, which turns out to be a drawback when extracting the relationship between two long separated words, self-attention mechanisms construct the relationship information between tokens by building an attention-matrix; this distributes proper weights to each token according to the relationship between tokens and importance of token information [20]. The transformer has achieved state-of-the-art performances in a variety of downstream tasks, resulting in a significant improvement in NLP. Nevertheless, it still relies on the training corpus that limits its utilization.

Recently, an advancement in NLP involved a novel self-attention based representation model namely bidirectional encoder representations from transformer (BERT), which was proposed by Google [21]. By pre-training on a large plain text corpus, the BERT model can focus on general human language understanding and distinguish among different use cases for a word [22]. In combination with fine-tuning of downstream tasks, BERT has achieved state-of-the-art results for a variety of NLP tasks [23]. In this way, recently modified versions of BERT-based models, such as Roberta [24], Albert [25], and Ernie [26] have enriched innovative methods in NLP, as they were developed through extensive large corpora such wikipedia and have been further optimized for model structure; therefore, they are worthy of investigation for NLP.

In the biomedical field, BERT models focusing on medical tasks were developed using large-scale biomedical corpora, such as ClinicalBERT [27] and BioBERT [28]. Additionally, recent studies have shown promising results using the BERT framework in the medical domain. Although an increasing number of Chinese BERT models have been released as open source and demonstrated state-of-the-art performance in NLP benchmarks [29–31], the research in Chinese clinical text data mining, especially in the analysis of radiology reports, is very limited compared with the global trend; this may be associated with barriers in accessing high quality report data and a lack of research pipelines [32]. The existing studies that use NLP techniques to classify actionable radiology reports are summarized in Table 1. To the best of our knowledge, there have been no attempts at using BERT and in-domain pre-training techniques in the classification tasks of Chinese actionable reports.

**Table 1:** Summary of NLP studies focusing on actionable radiology reports (ML: Machine Learning, DL: Deep Learning, BERT: Bidirectional Encoding Representation of Transformer).

Author(s)	Language	Number of radiology reports	Algorithm	Section of report	Research objective
Carrodeguas et al. [33]	English	2306	ML/DL	Impression	Classifying recommendation
Helibrun et al. [34]	English	851	Rule-based	Impression	Detecting critical finding
Lou et al. [35]	English	6000	ML	Not mentioned	Classifying recommendation
Esteban et al. [36]	English	3401	Software	Findings, impression	Classifying recommendation
Morioka et al. [37]	English	1402	Rule-based	Not mentioned	Classifying disease condition
Fu et al. [38]	English	1000	Rule-based ML/DL	Not mentioned	Classifying disease condition
Nakamura et al. [39]	Japanese	63646	BERT	Order, findings, impression	Detecting critical finding
Jujjavarapu et al. [40]	English	871	ML	Not mentioned	Classifying disease condition
Liu et al.. [15]	Chinese	1089	BERT/ML	Findings	Classifying disease condition
Zhang et al. [41]	Chinese	359	BERT Pre-training	Findings	Classifying disease condition
Zaman et al. [42]	English	1503	BERT Pre-training	Findings	Classifying disease condition
Liu et al.. [43]	English	594	BERT	Not mentioned	Classifying certainty
Proposed study	Chinese	5864	BERT Pre-training DL	Findings	Classifying disease condition

The first contribution of this study is a novel approach in Chinese actionable report classification using BERT, CNN, multilayer perceptron (MLP), bi-directional LSTM (Bi-LSTM) and Bi-LSTM-CNN. In addition, we comprehensively evaluated and compared the benefits of three recently proposed Chinese variants of the BERT model: chinese-roberta-wwm-ext(abbreviated as Roberta), mengzi-bert-base(abbreviated as Mengzi), and chinese-bert-wwm-ext(abbreviated as Bert-wwm-ext). Second, with the help of in domain pre-training techniques, we further improved the performance of the BERT model with respect to accuracy (i.e., F1-score and AUC); these results illustrate the potential of further improvement with additional pre-training. Third, we conducted extensive experiments using max sequence length as a hyperparameter in the model fine-tuning strategy; the results demonstrated better performance in tokenizing with a length of 128 and 512 compared with other lengths tokenizing methods. Overall, our results have identified key information distribution in Chinese radiology reports and could improve related NLP studies in health-related texts, especially in ultra-long text tokenizing.

## Materials and methods

### Study overview

Figure 1 presents the workflow of the study, which is mainly composed of five sections: (1) data collection and labeling, (2) data preprocessing and partition, (3) text representation and pre-training, (4) classifier training, and (5) performance evaluation comparison. In the discussion section, the perspective of this study and the future utilization of the proposed model is summarized.

### Data collection and labeling

We retrospectively collected the Electronic Healthcare Recording (EHR) data from patients who were admitted with tinnitus and received temporal bone CT examinations between September 2014 and December 2021 from a tertiary hospital in Beijing, all the radiology reports were written in Chinese and stored in PACS (Picture Archiving And Communication System) developed by DJ HealthUnion. Patients with the intention of a subsequent visit after initial treatment were excluded from the study as their reports may contain postoperative features.

In temporal bone imaging, the critical part is the identification of imaging findings, which fully covers the feature of lesions in imaging and varies greatly due to complexity of related diseases. In contrast, the impression section may not offer useful information in this task because imaging is insufficient to give a clinical diagnosis of tinnitus. Therefore, imaging finding blocks were segmented and used in this study. Additionally, all patients’ private information was removed from reports.

Reports were reviewed and manually labeled by two radiologists with at least three years of experience in temporal bone CT reports. The labeling criteria were based on the diagnosis framework of tinnitus by Cima et al. [44]. Three classes were manually labeled as follows: normal, tinnitus unrelated finding, or tinnitus related finding. The details of labeling criteria are listed in Table 2. Before the start of the experiment, a Kappa test was conducted to verify the consistency of labeling performance using a sample of 300, which eventually resulted in a Kappa value of 0.79. The details of the Kappa test are listed in Additional file 1: Table S1. In the labeling process, discrepancies were addressed by a senior expert to achieve consensus; the reports that were not eventually defined were excluded from the study.

**Table 2:** Data labeling criteria

Classification	Potentially clinically important findings	Label instruction
Normal finding (labeled as 0)	NA	The scenarios when all organs are described as normal
Irrelevant finding (labeled as 1)	**Bone:**	If any lesion is observed and should be reported; meanwhile, the clinician is confident that the image finding provided limited information for diagnosis of tinnitus.
	Any Degeneration	
	**Brain:**	
	-Brain degeneration	
	**Nose and Sinus:**	
	-Nasosinusitis (frontal sinus, sphenoid sinus, ethmoid sinus, maxillary sinus (except acute inflammation involving adjacent bone structures))	
	-Nasal turbinate hypertrophy	
	-Deviation of nasal septum	
	-Sinus cyst	
	**External/middle ear:** -	
	Chronic middle ear mastoiditis (except acute inflammation involving adjacent bone structures)	
	-Auditory canal cerumen	
	-Low middle cranial fossa	
Relative finding (labeled as 2)	**Bone:** -	If one or more image findings should be reported in detail, and lead to a certain diagnosis for further examination or clinical evaluation. Or the image finding addressed a need for urgent communication with clinicians for timely treatment. Since there is variability in language expression, the labeler’s judgment is used as reference.
	Sigmoid sinus bone wall deficiency	
	-Superior semicircular canal dehiscence	
	-Auditory ossicle abnormality	
	-Bone fracture	
	-High jugular fossa	
	**Brain:**	
	-Neoplasms	
	-Intracranial hemorrhage	
	-Cerebral infarction	
	-Cerebral herniation	
	**Nose and sinus cavity:**	
	-Neoplasms	
	-Nasosinusitis (morphologically altered bone or sinus tract obstruction)	
	**External/Middle ear:**	
	-Tympanic lesion (inflammation, neoplasm or perforation)	
	-Otosclerosis	
	-Cholesteatoma	
	-Other neoplasm observed within the imaging field	

**Fig. 2:**
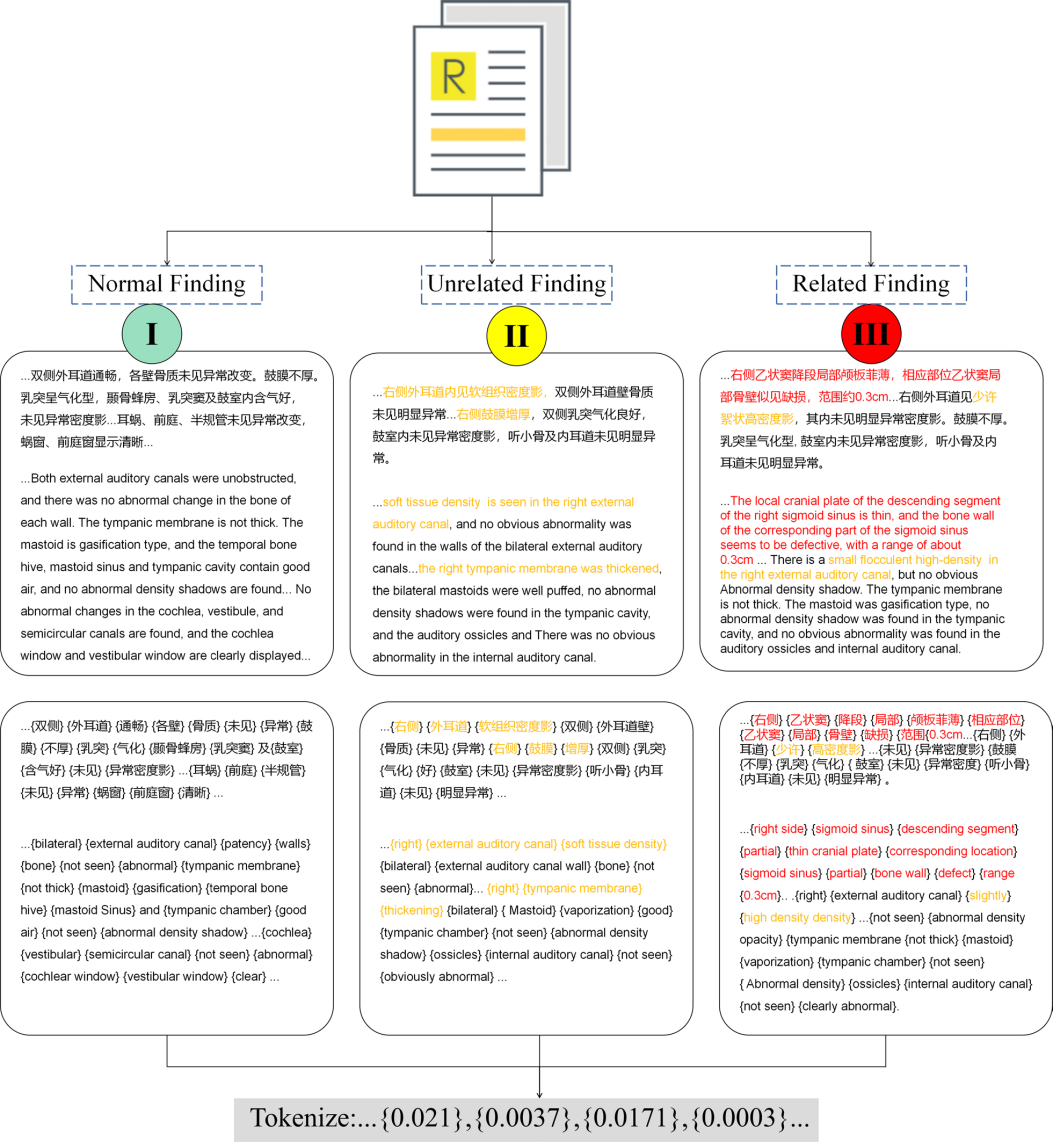
Details of report annotation and text preprocessing. Yellow characters represent findings irrelevant to tinnitus; red characters represent findings relevant to tinnitus and should be actionably reported in communication with physicians. *All reports were written in Chinese, and English translations are presented in the figure for illustration

After the screening, a total of 5864 reports were ultimately considered and annotated. They were then divided into 70% for training (n = 4104), 15% for validation (n = 880), and 15% for testing (n = 880) datasets. The label was controlled as a hierarchical indicator. The details for labeling and text preprocessing are illustrated in Figures 1 and 2.

### Data preprocessing

All reports were preprocessed before further modeling, the punctuations and linguistic tags were first removed from the text using a preprocessing pipeline. In unstructured radiology reports, although radiologists typically present information based on standard free-text templates of reporting, they may contain linguistic and computational symbols such as end-of-line (EOL), blank character (BC) or line break (LB). These are considered noisy data and lengthen the text; this phenomenon is more common when collecting data from long term historical datasets. Then, a Chinese stopword corpus is utilized to filter meaningless stopwords from the text; those words may contain Chinese auxiliary words used for literal sense of formality. Finally, comments that were noted in the report were removed from the text; the comments were used for internal communication between the workstation and radiologists to notify them of remarkable events in clinical workflow, and are not essential components of radiology reports.

### Text representation

Word embedding is an essential technique in NLP used to represent language based characters or words in quantitative values before further analysis. Typically, the embedded words could be represented in neighboring high-dimensional spaces according to the similarity of their actual meaning. Word embedding can be achieved through different embedding techniques, and each with its own pros and cons. In this study, we utilized two major methods for Chinese language embedding: Word2Vec and BERT.

#### Word2Vec as embedding method

Word2Vec is an algorithm that generates a high-dimensional vector according to the given input when accepting a certain training corpus; this means that words with similar literal meaning may stay in a relatively closer space to each other, and this feature enables the generated matrix to maintain certain information within the text. Furthermore, the Word2Vec algorithm also has shortcomings since it cannot accept new words if it is not included in the training process. In this study, we accepted one of the main architectures in the Word2Vec-Skip-Gram model as an embedding method, the details of embedding parameters is provided in  Additional file 1: Table S2.

#### BERT as embedding method

The BERT has been recently proposed by researchers in Google. The novelty of BERT is the application of the bi-layer transformer architecture - an attention-based mechanism that can accurately extract contextual relationships in words to realize unsupervised learning by combining text input and output through the decoder-encoder framework. The BERT was initially proposed after being trained on ultra large datasets such as Wikipedia, and an optimal performance may be achieved after fine-tuning datasets of downstream tasks.

### Classifier training

In our study, we used four recent state-of-the-art NLP classifiers: CNN, MLP, Bi-LSTM and a hybrid Bi-LSTM-CNN. As previously stated, CNN has the advantage of maximizing and extracting local features of neighboring words by convolutional and maxpooling layers, whereas Bi-LSTM models could store features of words in whole sentences by using cells and gates from both left and right directions to combine past and future context information from long-sentence radiology reports. The MLP model is a baseline deep learning model that is utilized for comparison, while the Bi-LSTM-CNN model is proposed as a hybrid neural network. After hyperparameter grid search, the optimum model performance is presented; the detailed description of those training parameters is listed in Table 3.

**Table 3:** Hyperparameters of model training

Model	Layers	Epochs	Batch size	Optimizer
CNN	16	20	32	Adam
MLP	16	20	32	Adam
Bi-LSTM	16	20	32	Adam
Bi-LSTM-CNN	32	20	32	Adam
BERT (variants) -fine tune	768	10	8	Adam
BERT-pre-training	768	10	8	Adam

### Fine-tuning of BERT-based models

As the BERT model can be applied to a variety of different natural language processing downstream tasks and only requires minimal adjustments, fine-tuning BERT with our labeled radiology reports has offered a great opportunity to exploit the advantages of the BERT framework and achieve competitive results. In the biomedical field, the fine-tuning technique has attracted much attention in classification tasks [21,45], however, the attempts in NLP tasks of Chinese radiology reports seems sparse. However, recently proposed novel variants of BERT-based Chinese embedding models have offered greater potential for further boosting NLP research in Chinese. To evaluate the benefits of BERT fine-tuning ,in the classification of tinnitus in Chinese radiology, BERT-base and the 3 variant models were used for fine-tuning in this stage.

Recently, many variants of BERT in Chinese have been published such as hfl/chinese-bert-wwm-ext (BERT-wwm-ext) and hfl/chinese-roberta-wwm-ext (Roberta) by Cui et al. [46], and Langboat/mengzi-bert-base (Mengzi) recently published by Zhang et al. [^47^]. These models were trained on large scale corpora, pre-trained with optimized strategy such as whole word masking, and achieved state-of-the-art (SOTA) performance in multiple official benchmarks such as GLUE, MNLI and QNLI. It is therefore attractive to testify the benefits of these models in Chinese medical domain tasks and evaluate their performance by fine-tuning. In this study, BERT-base-Chinese (BERT), hfl/chinese-bert-wwm-ext (BERT-wwm-ext), hfl/chinese-roberta-wwm-ext (Roberta), and Langboat/mengzi-bert-base (Mengzi) were enrolled in the framework and compared. Additionally, the hyperparameters of each model are provided in Additional file 1:Table S3.

**Fig. 3:**
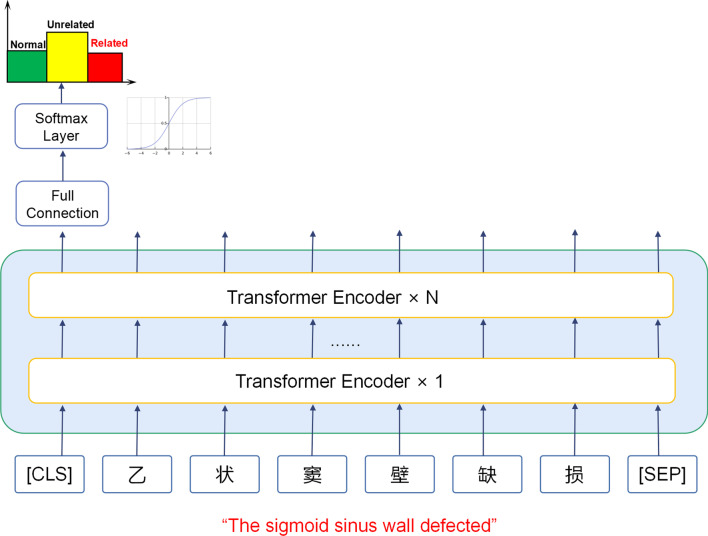
Illustration of architecture in BERT based models. The English subtitle is a translation of a sentence in a Chinese radiology report

For fine-tuning, one full-connection(FC) layer was added after BERT in combination with a softmax layer for the label output. For major hyperparameters, the max sequence length was set to 512, the training batch size was set to 16, and the training epoch was set to 10. The hyperparameters were chosen based on the memory and computing power of our GPU resources. We fine-tuned the mainstream BERT-based models in Chinese text NLP tasks: BERT-base-Chinese (BERT), hfl/chinese-bert-wwm-ext (BERT-wwm-ext) and hfl/chinese-roberta-wwm-ext (Roberta) along with Langboat/mengzi-bert-base (Mengzi). The basic architecture of BERT based models is illustrated in Fig. 3.

### In-domain pre-training of BERT

For further exploration of the potential of BERT-based models in language representation, and as it has been proven that pre-training can effectively improve model performance with limited data, we hypothesize that an in-domain pre-training task (IDPT) could be considered as a way for greater utilization of BERT in this task. The IDPT could be regarded as a process of transferring learning to integrate the domain-specific knowledge into the original BERT model, in this way, the initial weights of BERT could be adjusted adequately to maximize performance and accuracy in domain specific tasks [48]. Hence, in order to transfer the domain-specific knowledge and language representation of tinnitus to form a domain-specific healthcare BERT model, we used a large-scale database of tinnitus related clinical notes for IDPT of BERT.

In the IDPT stage, we collected 3873 clinical cases and 1431 radiology reports, which accounts for about 1 million words. For the preprocessing stage, the space and newline symbols were removed from the corpus to form the corpus data; thereafter, no further processing was performed. The pre-trained tinnitus-BERT model was trained in a way described in literature [48], the hyperparameters, computing resource and training time in the IDPT stage is listed in Table 4.

**Table 4:** Training time, computing resource and hyperparameters in IDPT

Data size	Train epochs	Train batch_size	Eval batch_size	Eval strategy	Eval steps	GPU	Pre-training time per epoch
10.7 MByte	10	16	16	Steps	100	Nvidia GTX1070	32 min

### Token length optimizing strategy (TLOS) based on max sequence length

**Fig. 4:**
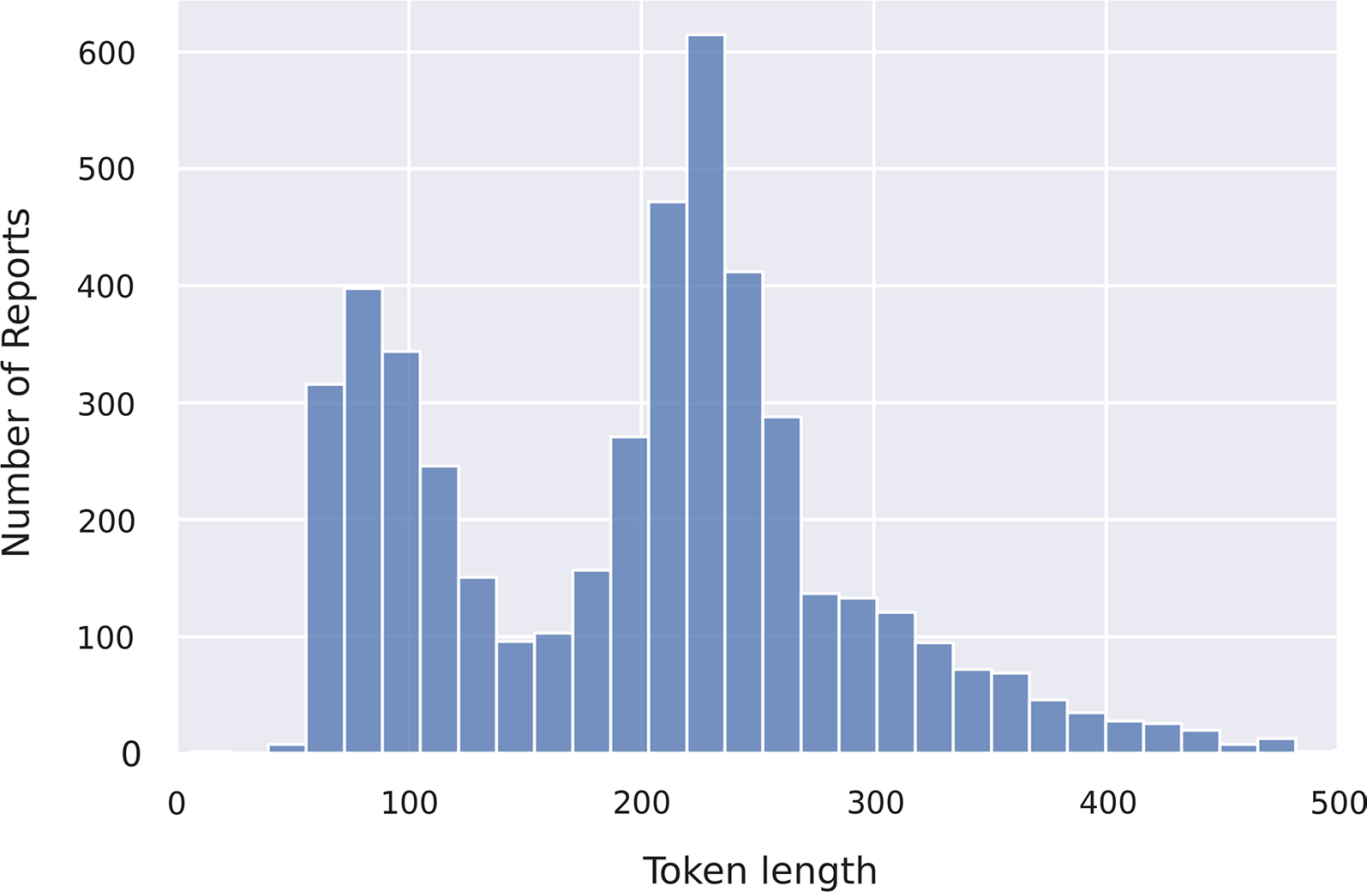
Token length distribution in training dataset

Max sequence length is a critical hyperparameter in the BERT model. For long sequence embedding, the token overflowing the max sequence length would be cut, while short sequences would be “padded” (i.e., filled with zeros or specific number) to the same length; this mechanism aims for constant length alignment of all input text. The Chinese-based BERT models use each character as a token; however, the token length of sequences in within each group varies widely. Figure 4 shows the disparity with two “peaks” along with the “long tail” in token distribution number, whereas the statistics in Table 5 show this variation more precisely. Previous studies have suggested that report length is affected by the amount of confidence the radiologist has in their analysis, we hypothesize that the token length in this study may lead to further investigation of patterns of Chinese radiology reports[49].

**Table 5:** Token length distribution in all training datasets.

	Label	Average token length (±standard deviation)	Number of samples
Normal finding	0	182.92±12.62	1100
Unrelated finding	1	237.73±28.45	2851
Related finding	2	262.52±47.13	1913

This phenomenon may be caused by the following reasons: (1) for patients with multiple or complex lesions, detailed reporting of radiological manifestation is needed, and many radiologists typically write an individual section of the foremost imaging findings before all findings for timely attention, thus prolonging the whole report; (2) the standards of reporting across historical timelines may have changed, as the EHR system may have progressed with further requests for more detailed reporting; (3) there is a variance in the writing style of different radiologists, particularly considering the differences in experience and skills.

A long sequence would consume more GPU memory and computational resources, especially when deploying large models such as BERT[50], and clinical departments would not commonly deploy high performance GPUs and RAMs. To address this uncertainty, we hypothesize that the foremost section of radiology reports be considered as a priority in classification; this could be testified by using max sequence length as a variable to evaluate the performance of models in encoding. In this experiment, we applied the sequence length values of 128, 256, 328, 468 and 512 (default), and compared the results to test our conjecture.

## Results

### Evaluation method

The performance of each method was evaluated using the receiver operating characteristic (ROC) curve, along with accuracy, precision, recall and F1-score. Further, true positive (TP) and false positive (FP) are the number of positive cases correctly and incorrectly predicted, while true negative (TN) and false negative (FN) are the number of negative cases correctly and incorrectly predicted. Equations 1 (1)-(4) describe the performance metrics, and the confusion matrix of results is presented in Additional file 1: Table S4.

1 $$\mathrm{Accuracy}=\frac{\mathrm{TP}+\mathrm{TN}}{\mathrm{TP}+{\mathrm{TN}}+{\mathrm{FP}}+{\mathrm{FN}}}$$

2 $$\mathrm{Precision}=\frac{\mathrm{TP}}{\mathrm{TP}+\mathrm{FP}}$$

3 $$\mathrm{recall}=\frac{\mathrm{TP}}{\mathrm{TP}+\mathrm{FN}}$$

4 $${\hbox{F}}1{\text{-score}}=\frac{2}{1/{\hbox{precision}}+1/{\hbox{recall}}}$$

Eqs 1: Equation for performance metrics

### Experiment 1:BERT finetuning model in comparison with Word2Vec based deep learning models

**Fig. 5:**
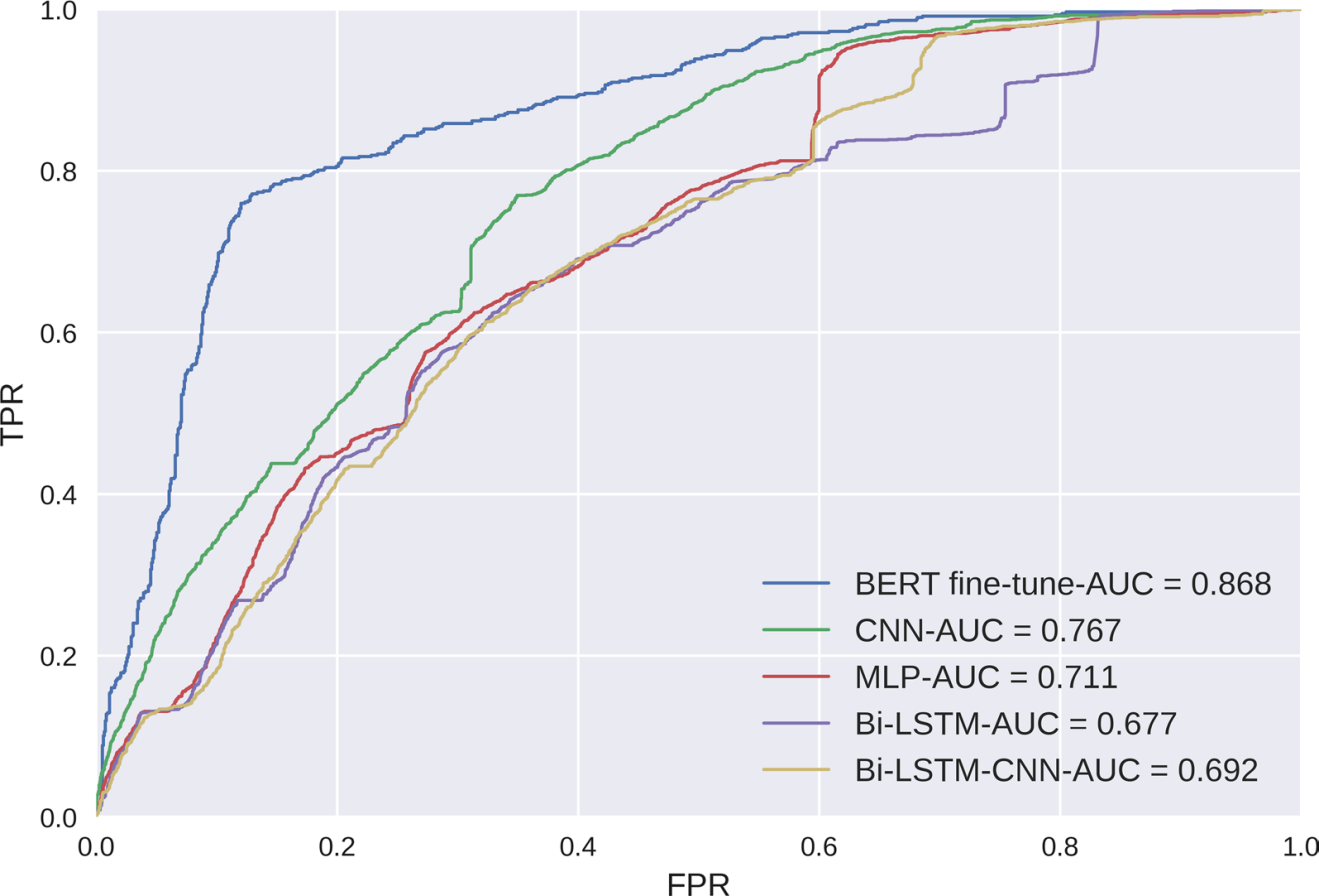
ROC curve and AUC values of BERT fine-tune and deep learning models. FPR:False Prediction Rate; TPR: True Prediction Rate.

**Fig. 6:**
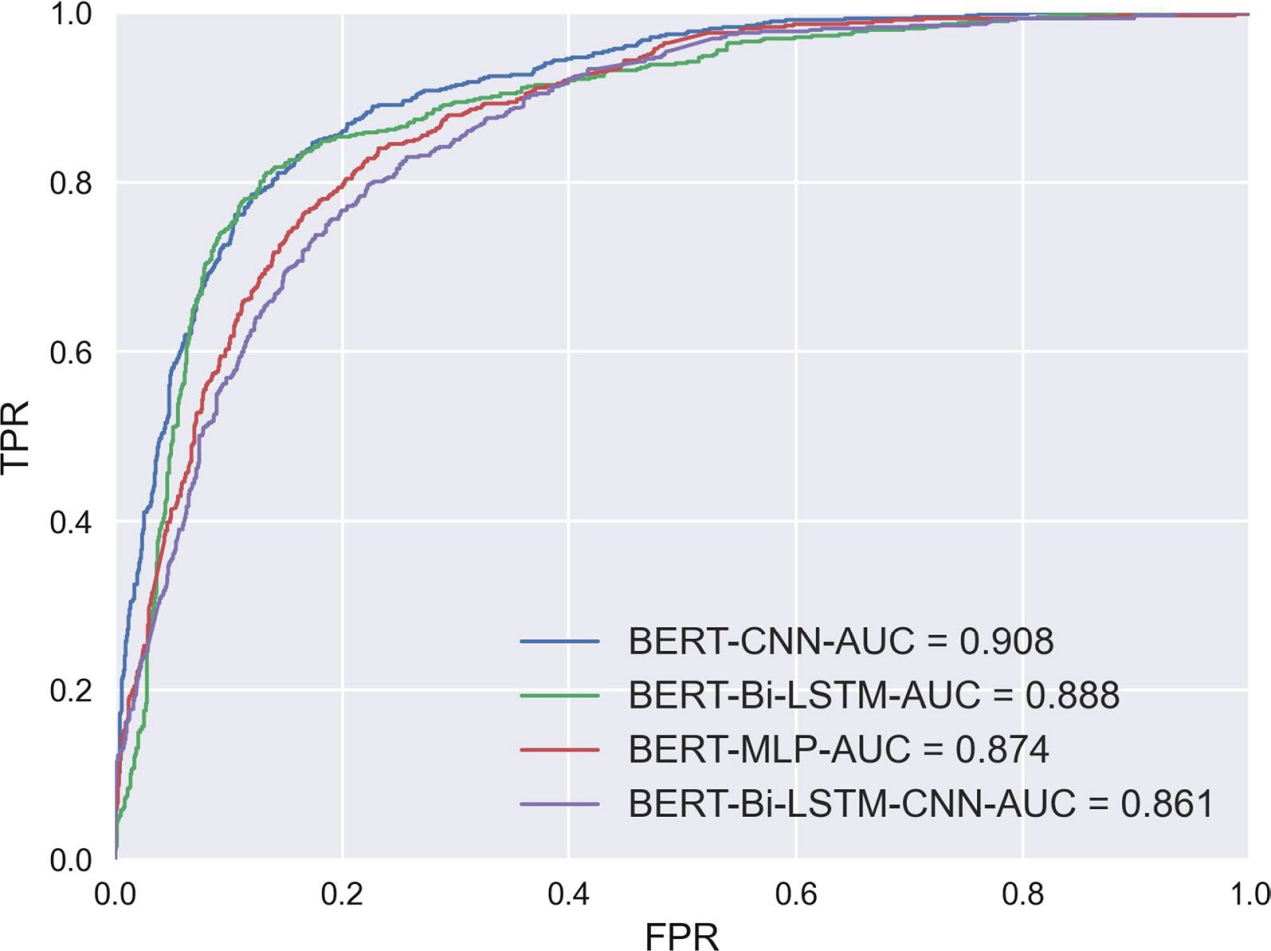
ROC curve and AUC values of BERT combined with deep learning models. FPR:False Prediction Rate; TPR: True Prediction Rate.

As a main goal of this study is to test the benefit of using BERT fine-tune in radiology classification task, the first experiment compares the results of the proposed Word2Vec embedding and classifiers: CNN, MLP, Bi-LSTM, Bi-LSTM-CNN with the BERT-based fine-tuning approach for classifying normal, tinnitus unrelated, and tinnitus related actionable radiology reports using collected data. The results are shown in Tables 6, 7, and the ROC curve is shown in Figure 5. The BERT fine-tune model outperformed the Word2Vec based models, BERT fine-tune achieved both the highest AUC of 0.868 and F1-score of 0.760; however, the difference between BERT fine-tune and second highest model (i.e., Word2Vec+CNN) is not larger than 1%. In addition, the approaches which combined BERT with classifiers were also evaluated, and the results are shown in Figure 6.

**Table 6:** Training Time and Hyperparameters in TLOS

Token length	Train epochs	Batch size	Optimizer	Learning rate	Training time per epoch (Min)
128	10	16	Adam	2e-5	12±0.24
256	10	16	Adam	2e-5	23±0.70
328	10	16	Adam	2e-5	31±0.47
468	10	16	Adam	2e-5	39±1.21
512	10	16	Adam	2e-5	43±0.62

**Table 7:** Comparison of model performance metrics

Embedding	Classifier	Accuracy	Precision	Recall	AUC	F1-score
Word2Vec	CNN	0.729	0.744	0.729	0.767	0.733
	MLP	0.644	0.643	0.644	0.711	0.644
	Bi-LSTM	0.737	0.740	0.737	0.677	0.738
	Bi-LSTM-CNN	0.728	0.729	0.728	0.692	0.728
BERT	CNN	0.770	0.788	0.777	0.908	0.781
	MLP	0.719	0.714	0.719	0.874	0.712
	Bi-LSTM	0.777	0.792	0.780	0.888	0.774
	Bi-LSTM-CNN	0.698	0.696	0.698	0.861	0.690
	Fine-tune	0.760	0.761	0.759	0.868	0.760
	IDPT	**0.842**	**0.843**	**0.842**	**0.948**	**0.841**
BERT-wmm-ext	Fine-tune	0.756	0.756	0.756	0.883	0.754
Mengzi	Fine-tune	0.751	0.751	0.751	0.846	0.750
Roberta	Fine-tune	0.767	0.767	0.767	0.878	0.764

The highest index is highlighted in bold

### Experiment 2: evaluation of BERT fine-tune and BERT in-domain pre-training(IDPT) and comparison with BERT original

**Fig. 7:**
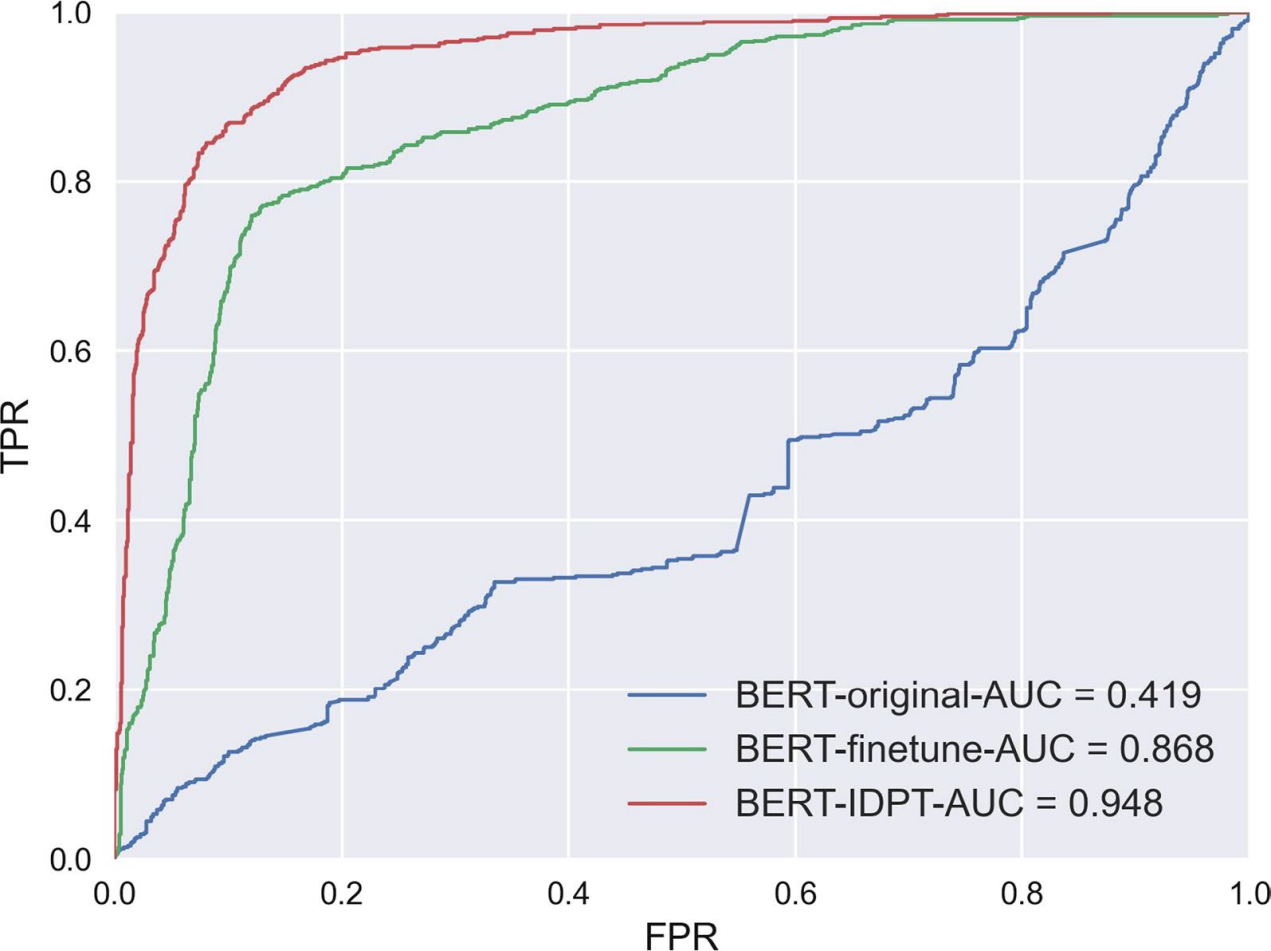
ROC curve and AUC values of BERT original, BERT-finetune and BERT-IDPT model.FPR:False Prediction Rate; TPR: True Prediction Rate.

The IDPT technique is applied in this study to further exploit the advantage of using BERT frameworks in pre-training. We compared the performance of the classification task certainty using three stages of BERT models: BERT original,BERT fine-tune and BERT-IDPT. The ROC curve and AUC values are shown in Figure 7 and the metrics are shown in Table 7. Compared to the results of BERT fine-tune, the BERT IDPT model obtained a significant improvement with AUC of 0.948 and F1-score of 0.841. In addition, the results demonstrated that after fine-tuning, the BERT model was efficiently adjusted to fit the task, when compared with original state, the AUC of the BERT fine-tune model increased from 0.419 to 0.868.

### Experiment 3: Evaluation of BERT Variants models with fine-tuning

**Fig. 8:**
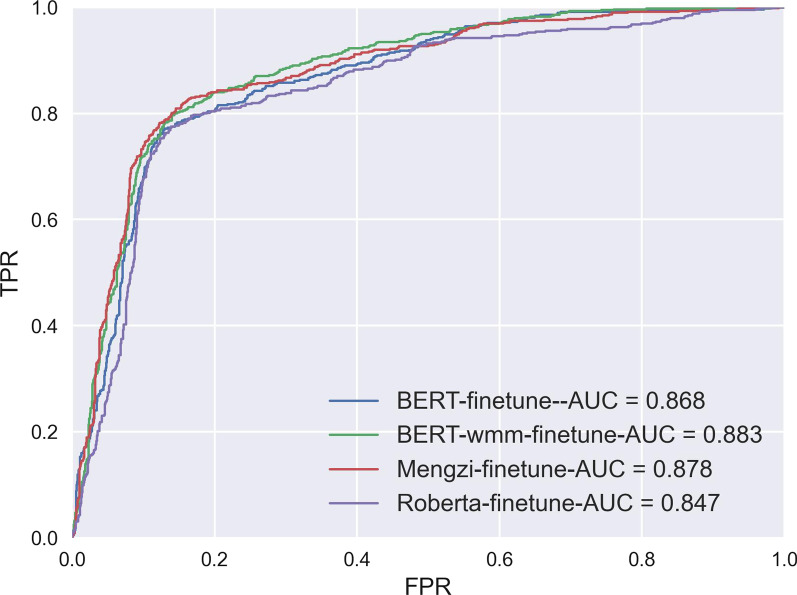
ROC curve and AUC values of BERT and Variant models. FPR:False Prediction Rate; TPR: True Prediction Rate.

To evaluate the benefits of using novel BERT-variant models in Chinese radiology reports classification, we compared the results of the four models after fine-tuning, the ROC curve and AUC values are shown in Figure 8 and the metrics are shown in Table 7. As a result, the Mengzi-model yielded the best AUC of 0.878 and F1-score of 0.764. Meanwhile, Roberta and BERT-wwm-ext achieved a relatively equal score compared to the BERT model. In general, the results are promising but not supportive to demonstrate a comprehensive improvement.

### Experiment 4: token length optimizing strategy (TLOS) based on max sequence length

**Fig. 9:**
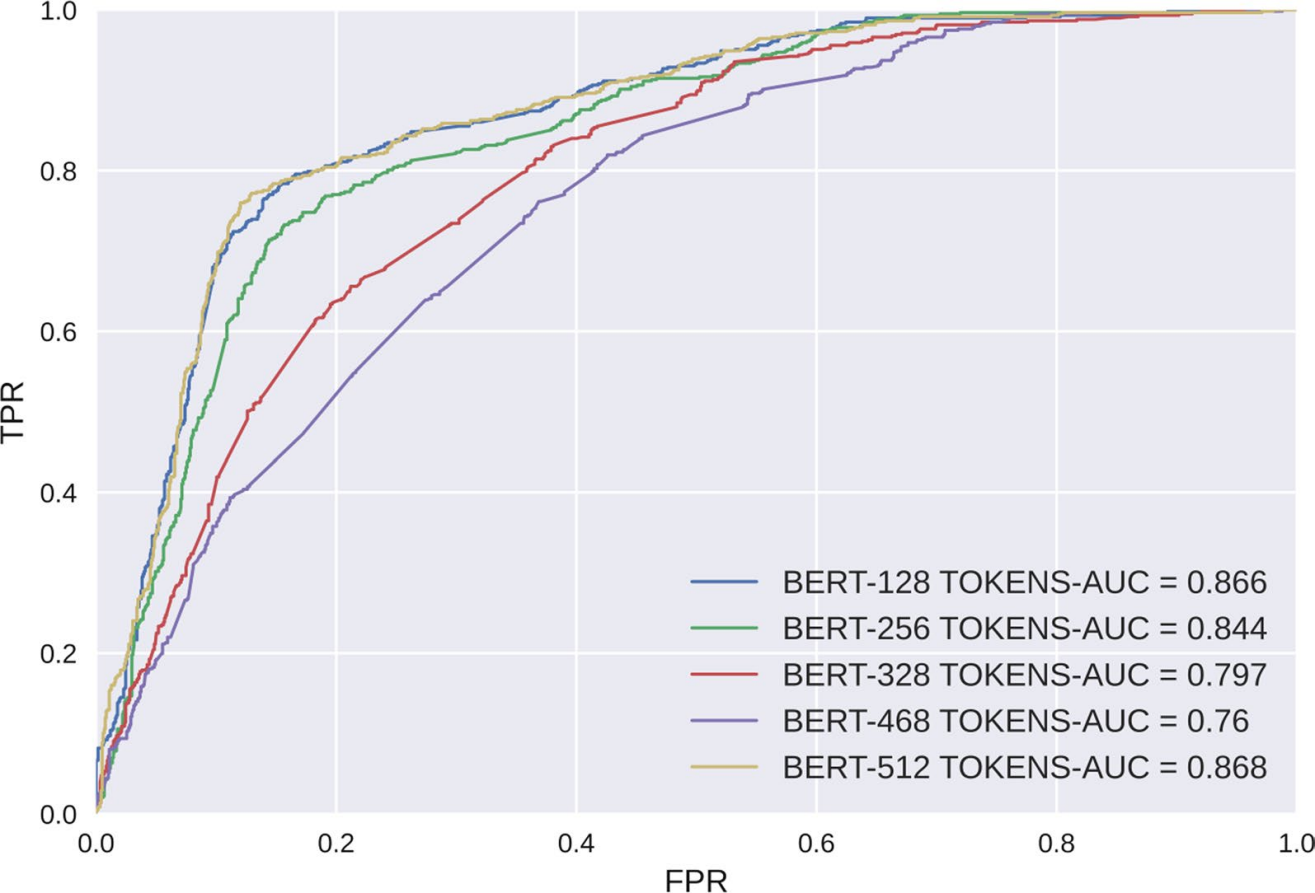
ROC curve and AUC values of the BERT model using different max sequence lengths. FPR:False Prediction Rate; TPR: True Prediction Rate.

**Fig. 10:**
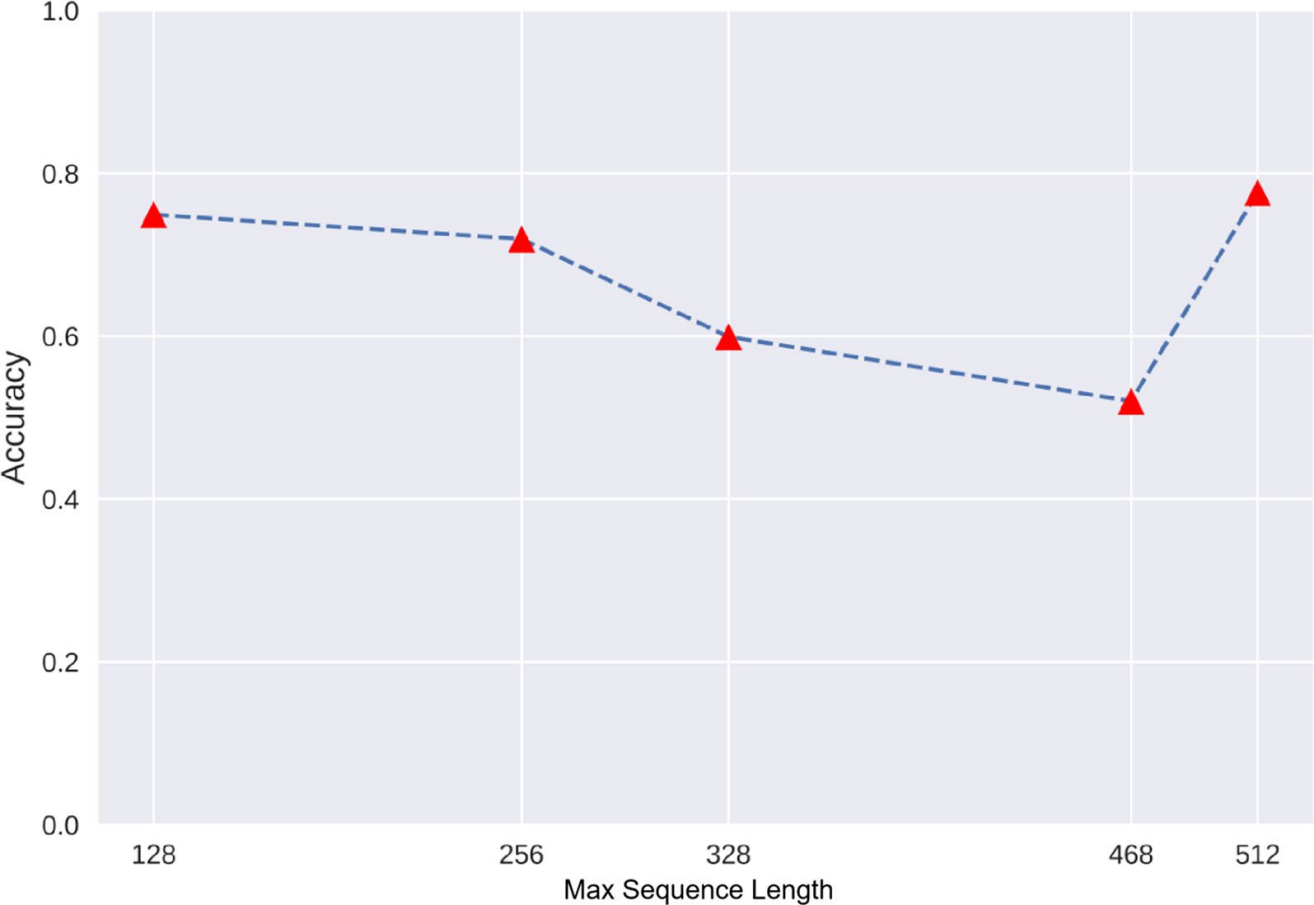
Comparison of accuracy in the BERT model using different max sequence lengths

As a result, the BERT model with full sequence length (512) achieved the highest AUC value and F1-score but also cost the longest training time-43 minutes per epoch. However, we noticed that the second highest score was yielded for the shortest sequence length (i.e., 128-token group), with a relatively equal AUC score of 0.868 (versus 0.878 in the 512-token group); also in addition, the F1-score in the 128-token group was 0.736 compared with 0.760 in the 512-token group. However, the accuracy declined when the sequence length increased from 128 to 468, with the lowest score in the 468-token group. The metrics are shown in Table 8, the Training Time and Hyperparameters in TLOS are descripted in Table 6, the ROC curve and AUC values are shown in Figure 9, and the relationship between accuracy and token length is shown by a dot plot in Figure 10.

**Table 8:** Comparison of BERT finetune with different max sequence lengths

Model	Max sequence length	Accuracy	Precision	Recall	AUC	F1-score
BERT fine-tune	128	0.741	0.738	0.741	0.866	0.736
	256	0.71	0.707	0.71	0.843	0.708
	328	0.616	0.627	0.616	0.797	0.601
	468	0.551	0.557	0.551	0.759	0.546
	512	0.760	0.761	0.759	0.868	0.760

## Discussion

Radiology reports are an essential component of big medical data. Previous studies have fully demonstrated the feasibility of extracting evidence from radiology reports to assist clinical diagnosis and prognosis and promote automatic communication between physicians, radiologists and patients[37,39,42].However, the full potential of NLP remains to be further discovered, whereas deep learning-based algorithms have nearly revolutionized the paradigm of medical imaging. Radiology reports are primarily intended to provide information to assist with diagnosis; this information must be interpreted by physicians before being transmitted to patients. However, this may not be guaranteed because of the busy schedules of physicians and lack of expertise who are knowledgeable about tinnitus diagnosis and treatment, which can negatively affect doctor-patient interactions and potentially adversely impact patient outcomes[51]. In addition, there is still controversy regarding the appropriate imaging of tinnitus[52]. Despite the consensus declaration of multiple medical societies[1,4,44], large-scale real-world evidence for quantifying the effectiveness of imaging results is urgently needed to justify their opinions. Therefore, it is necessary to promote the research into the application of NLP-based technology to tinnitus radiology reports.

The language representation method is one of the highlights of NLP studies. Recent NLP studies in actionable radiology reports include two types of approaches: (1) rule/pattern-based framework, and (2) deep learning/BERT-based framework. Many studies that have used the former technique report promising results with pre-defined patterns while having poor generalization ability in other tasks. Meanwhile, BERT-based approaches have gradually performed well with more skillful fine-tuning and pre-training strategies. However, the studies focusing on Chinese radiology reports are rare.

From the same starting point, Aaron et al. reported a machine learning-based classification of temporal bone imaging reports for the identification of inner/middle/outer and mastoid abnormalities[53]. Although this method has achieved good results, it fails to classify the abnormal patients with clinical significance. In contrast to previous studies that use BERT to classify actionable radiology reports with term-specific strategy or covering multiple pathological characteristics, our study has three novelties: (1) a framework of fine-grained labeling strategy to improve practical value in clinical scenarios, (2) the utilization of a relatively large disease-specific corpus in-domain pre-training strategy to improve the model performance; and (3) the feasibility of using shorter sequence length to accelerate model building while maintaining its performance. These innovations may contribute to further use of BERT in Chinese medical text analysis through NLP technology.

In the first experiment, we demonstrated the benefit of using BERT compared to other deep learning models including Bi-LSTM, CNN and hybrid Bi-LSTM-CNN. Although the results did not show large difference in F1-score (BERT:0.760, CNN: 0.733), BERT fine-tuning achieved a higher AUC value of 0.866(CNN:0.767). In experiment 2, we further used transfer learning in BERT by pre-training an in-domain corpus that elevated the F1-score (BERT: 0.760 versus BERT-IDPT: 0.841) and AUC (BERT: 0.868 versus BERT-IDPT:0.948); this indicated competitive performance in the classification task.

Pre-training is an important technique in NLP field, this approach has recently attracted increasing attention, especially in healthcare related fields. For instance, Zhang et al. [41] designed and evaluated the feasibility of using pre-training models to extract key information from Chinese radiology reports fort lung cancer staging, the model achieved an F1 of 85.96%,while our study achieved an F1 of 84.10%. More recently, Nakamura et al. [39] applied BERT without IDPT to classify actionable Japanese radiology reports, and attempted to predict a positive/negative “actionable tag”, the results seem promising with highest AUC of 0.95. In comparison with previous studies on radiology report classification, the labeling methods applied in this study were more complex, which require both physicians’ clinical experience and priori anatomic knowledge of radiology. Moreover, we utilized IDPT to the improve the BERT model with domain specific knowledge, which has reported to be state-of-the-art performance.

Finally, we define this study as customized research with practical purpose, considering that the large computational demand of BERT in long sentence processing may not be fully satisfied under common deployment situations. Based on the authors’ working experience as radiologists, we propose a max sequence token adaptation strategy to assess the performance with partial embedding. The results showed that the 128-token embedding achieved a relatively equal performance compared with whole sentence embedding (F1-score of 0.736 in 128 tokens versus 0.760 in 512 tokens, AUC of 0.866 in 128 tokens versus 0.868 in 512 tokens). This result may be partially explained by the tacit occupational habit of radiologists to record the most emergent finding in an individual paragraph before normal findings.

Lastly, this study has several limitations that need to be discussed. First, although the data size of this study (5864 reports for training, 3873 clinical cases and 1431 radiology reports for in domain pre-training) is relatively large compared to related studies (presented in Table 1), the bias should be considered as it is a single-center study. More data from multiple centers and bias correction may enable more efficient transfer learning of BERT to yield promising results in real world scenarios, which we will pursue in the near future. Second, we developed and evaluated the BERT-based framework to identify actionable radiology reports from temporal bone imaging. However, the generalizability of this model to other types of radiology reports, such as head CT, MRI, and so on needs to be further evaluated with more fine-tuning strategies. Furthermore, it is worth noting that our proposed framework is a semi-automated pipeline that requires no further remodeling of the base architecture. In this regard, physicians with clear purpose of research demand should benefit by merely focusing on the labeling criteria. Third, the hyperparameters of this model, such as batch size, training epoch or learning rate are limited by computing resources. To pre-train a wider range of data and realize more comprehensive results, a more advanced operating environment would be necessary. Some examples could be ClinicalBERT, which was trained by 2,000,000 clinical notes from the MIMIC-III database and BioBERT that was trained using all PubMed publications.

## Conclusion

In this study, we proposed a BERT based framework using an in domain pretraining technique to classify actionable radiology reports in tinnitus patients. The experimental results show that our model outperforms the benchmark deep learning base models, BERT-base model and BERT variants. Additionally, we proposed a max-sequence-length adaption method for processing long text Chinese radiology reports. This study may promote the using of BERT in clinical decision support and academic research.

## Supplementary Information

**Additional file 1.** Related information of data analysis and model-construction for this paper.

## Data Availability

The datasets generated during and analyzed during the current study are not publicly available due to the institution’s policies involved in Human genetics resources, but are available from the corresponding author on reasonable request. The code used during the current study are available in https://github.com/currylee92/BERT.

## Declarations

### Accordance Statement

All methods were performed in accordance with the relevant guidelines and regulations.
