# Supplementary material for: Automatic text classification of actionable radiology reports of tinnitus patients using bidirectional encoder representations from transformer (BERT) and in-domain pre-training (IDPT)
Source: BMC Med Inform Decis Mak. 2022 Jul 30;22:200. doi: 10.1186/s12911-022-01946-y (PMC9338483; doi:10.1186/s12911-022-01946-y)

**Supplementary Materials:**

**Figure S1 Example of Basic structure of Radiology Report**


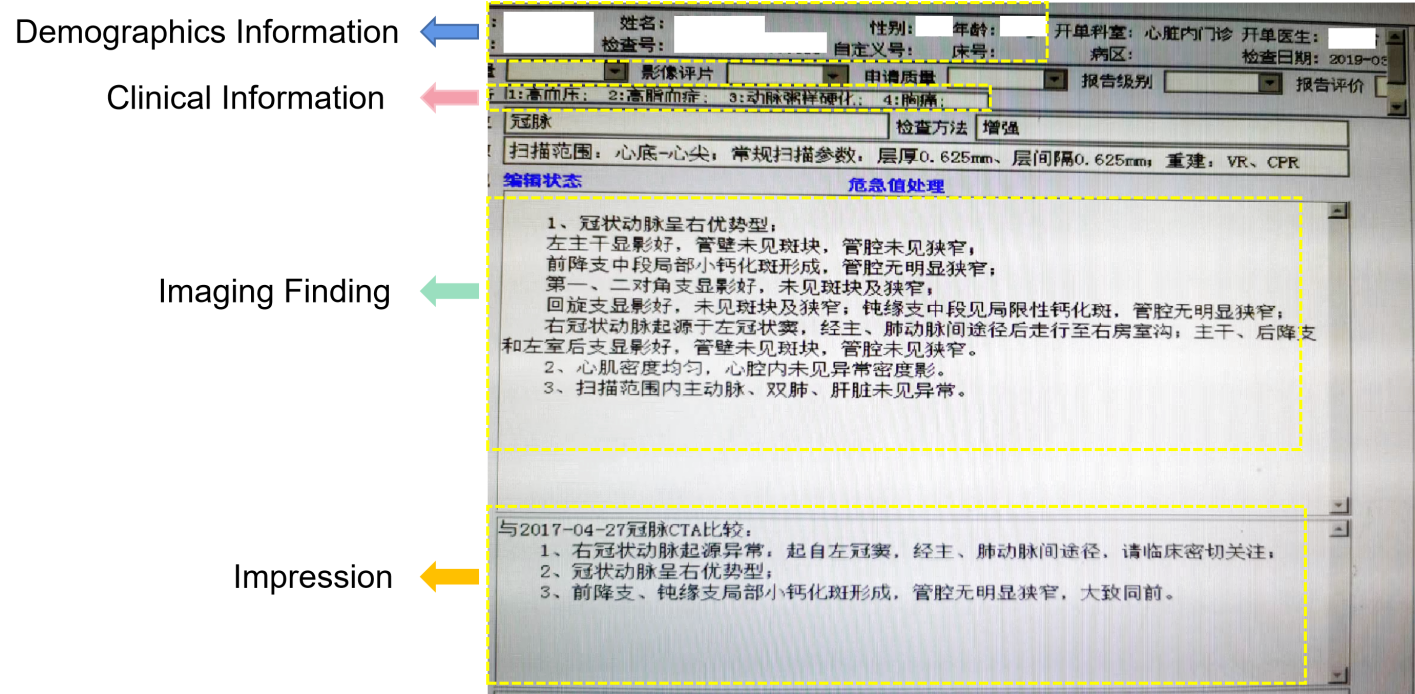


**Table S1: Kappa results of labelers (n=300)**

| **Labeler 1**  **Labeler 2** | **Normal Finding** | **Unrelated Finding** | **Related Finding** | **Total** |
| --- | --- | --- | --- | --- |
| **Normal Finding** | **92** | 5 | 0 | 97 |
| **Unrelated Finding** | 10 | **88** | 6 | 104 |
| **Related Finding** | 1 | 19 | **79** | 99 |
| **Total** | 103 | 112 | 85 | **300** |

Pe = (103*97+112*104+85*99)/300*300=0.333

Pa =（92+88+79）300=0.863

Kappa =（Pa-Pe）/（1-Pe）=0.795

**Eqs2:Equation for performance metrics**

**Table S2: Word2Vec Parameters**

| **Parameters** | **Value** |
| --- | --- |
| **Sg** | 1（skip-gram） |
| **Size** | 100 |
| **Alpha** | 0.025 |
| **Sample** | 1e-3 |
| **Max_vocab_size** | None |
| **Min_Alpha** | 0.0001 |
| **Iter** | 5 |
| **Batch_Words** | 10000 |

Table S3: BERT Parameters

| **Model** | **Size** | **Hidden_Size** | **Num_Attention_Heads** | **Num_Hidden_Layers** | **Learning**  **Rate** | **Epoch** | **Batch_Size** |
| --- | --- | --- | --- | --- | --- | --- | --- |
| **Bert-base-chinese** | 393MB | 768 | 12 | 12 | 2e-5 | 10 | 8 |
| **Chinese-Bert-Wwm-Ext** | 390MB | 768 | 12 | 12 | 2e-5 | 10 | 8 |
| **Chinese-Roberta-Wwm-Ext** | 390MB | 768 | 12 | 12 | 2e-5 | 10 | 8 |
| **Mengzi-Bert-Base** | 196MB | 768 | 12 | 12 | 2e-5 | 10 | 8 |

**Table S4: Statistics Of Token Length**

| Count | Mean | Std | Min | 5% | 25% | 50% | 75% | 80% | 95% | Max |
| --- | --- | --- | --- | --- | --- | --- | --- | --- | --- | --- |
| 5864 | 186.02 | 71.22 | 16.00 | 58.00 | 133.00 | 247.00 | 306.00 | 328.00 | 468.00 | 512 |

**Table S5** Confusion Matrix


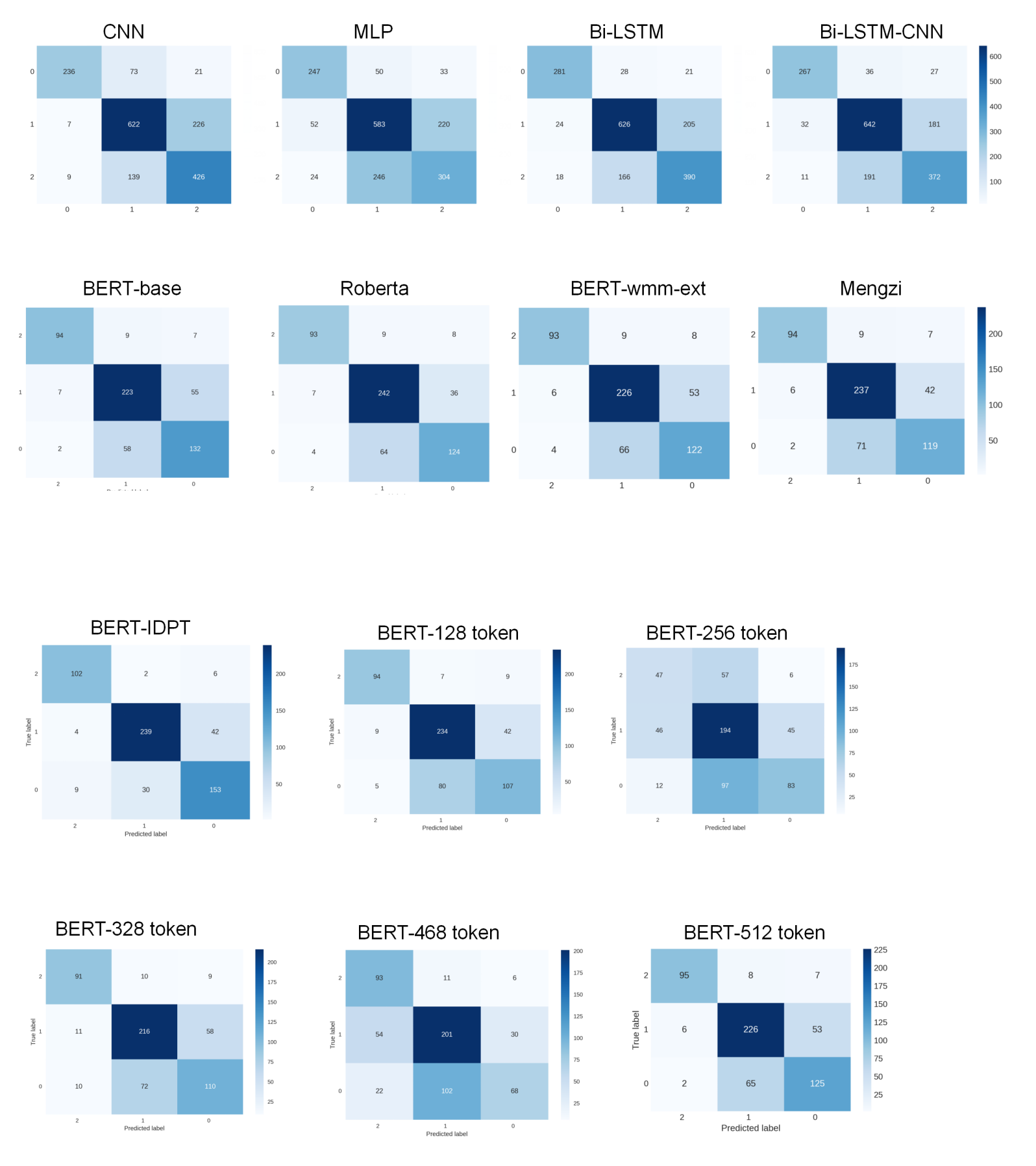

Supplement: Supplementary file 1 — Additional file 1. Related information of data analysis and model-construction for this paper. [file 12911_2022_1946_MOESM1_ESM.docx]
